# Carbapenemase-producing *Enterobacteriaceae*, U.S. Rivers

**DOI:** 10.3201/eid1102.030684

**Published:** 2005-02

**Authors:** Cécile Aubron, Laurent Poirel, Ronald J. Ash, Patrice Nordmann

**Affiliations:** * University Paris XI, Paris, France;; † Washburn University, Topeka, Kansas, USA

**Keywords:** resistance, imipenem, beta-lactamase, *Enterobacteriaceae*, environment, research

## Abstract

Our study was initiated by previous isolation of 30 imipenem-resistant, gram-negative rods from 7 of 16 U.S. rivers sampled from 1999 to 2001. Imipenem hydrolysis was detected in 22 of those isolates identified as *Enterobacter asburiae*. Random amplified polymorphism DNA analysis showed that these *E. asburiae* isolates were genetically indistinguishable. An identical clavulanic acid–inhibited β-lactamase IMI-2 was identified from each isolate that shared 99% and 97% amino acid identity with the chromosome-encoded β-lactamases IMI-1 and NmcA, respectively, from *E. cloacae* clinical isolates. The *bla*_IMI-2_ gene was located on a self-transferable 66-kb plasmid. Sequence analysis of a cloned 5.5-kb DNA fragment obtained from 1 of the imipenem-resistant *E. asburiae* isolates identified an upstream LysR-type regulator gene that explained inducibility of IMI-2 expression. β-Lactamase IMI-2 is the first inducible and plasmid-encoded carbapenemase. Identification of clonally related *E. asburiae* isolates from distant rivers indicates an environmental and enterobacterial reservoir for carbapenemase genes.

## Abstract 2

Identification of imipenem-resistant *Enterobacter asburiae* isolates from distant rivers indicates an environmental reservoir for carbapenemase genes.

Carbapenems, such as imipenem and meropenem, are the most potent β-lactam antimicrobial drugs for avoiding resistance in gram-negative rods. Resistance to carbapenems is rare in *Enterobacteriaceae* and may be mediated by 3 mechanisms: hyperproduction of an AmpC-type cephalosporinase combined with decreased drug permeability through the outer membrane, decreased affinity of penicillin-binding proteins that constitute target proteins for carbapenems, and carbapenem-hydrolyzing β-lactamases (*1**–**3*). These rare carbapenemases may be either plasmid-mediated metallo-β-lactamases (IMP- and VIM-type) or chromosomally encoded and clavulanate-inhibited enzymes (NmcA, IMI-1, Sme-1/Sme-2) (*2**,**4**–**9*). The latter group of enzymes shares consistent percentage of identity and belongs to the Ambler class A of β-lactamases (*2**,**10*). Very recently, plasmid-mediated and clavulanate-inhibited carbapenemases have been reported as a source of nosocomial infections in U.S. hospitals (*11**–**15*).

While the role of animals in the emergence of clinically important, antimicrobial-resistant strains has been extensively shown (e.g., in *Salmonella* spp.), the role of aquatic environment as a reservoir of antimicrobial-resistance genes is less established (*16**–**21*). A recent study described high levels of antimicrobial-resistant strains from U.S. rivers (*22*). We identified the imipenem-resistant, gram-negative strains recovered from that study and analyzed the molecular mechanism involved in carbapenem resistance of the imipenem-resistant enterobacterial strains. Clonally related *Enterobacter asburiae* strains were identified in midwestern U.S. rivers. *E. asburiae* naturally produces a cephalosporinase but no carbapenemase and may be responsible for nosocomial infections (*23*). Here, the strains expressed a novel plasmid-encoded and clavulanate-inhibited carbapenemase.

## Materials and Methods

### Bacterial Isolates

A previous study identified 30 imipenem-resistant, gram-negative strains out of 1,861 ampicillin-resistant, gram-negative isolates from 7 out of 16 U.S. rivers that were sampled from 1999 to 2001 (*22*). Identification of these imipenem-resistant isolates was performed by conventional biochemical techniques (API-20E and API-NE systems [bioMérieux, Marcy-l'Etoile, France]), and confirmed by 16S rDNA sequencing (*24*).

*E. asburiae* CIP 103358 and *E. asburiae* CIP 105006 were used as reference strains (Institut Pasteur strain collection, Paris, France). *E. cloacae* NOR-1 and *E. cloacae* 1413B were used as strains that produce the chromosome-encoded, clavulanate-inhibited carbapenemases NmcA and IMI-1, respectively (*5**,**8*). One of the *E. asburiae* isolates recovered from a river (strain MS7) was used for cloning experiments. Streptomycin-resistant *Escherichia coli* DH10B strain was used in cloning and conjugation experiments (Life Technologies, Eragny, France).

### Antimicrobial Agents and Resistance Study

The antimicrobial agents and their sources were as follows: amoxicillin, ceftazidime, clavulanic acid, and ticarcillin (GlaxoSmithKline, Nanterre, France); aztreonam (Bristol-Myers Squibb, Paris La Defense, France); cephalothin (Eli Lilly, Saint-Cloud, France); piperacillin and tazobactam (Lederle, Les Oullins, France); cefotaxime (Aventis, Romainville, France); imipenem (without cilastatin) (Merck Sharp and Dohme, Paris, France); meropenem (AstraZeneca, Paris, France); ampicillin and streptomycin (Sigma, Paris, France).

MICs were determined by an agar dilution technique on Mueller-Hinton (MH) agar (Sanofi Diagnostics Pasteur, Marnes-La-Coquette, France) with an inoculum of 10^4^ CFU per spot (*25*). Carbapenemase activity was determined by UV spectrophotometry with culture extracts of each of the imipenem-resistant, gram-negative rods and imipenem (100 µmol) as substrate, as reported previously (*26*). One unit of enzyme activity corresponded to the hydrolysis of 1 µmol of substrate per min. Inducibility of the β-lactamase expression was determined with imipenem and cefoxitin as β-lactamase inducers, as described (*27*). Briefly overnight culture of each imipenem-resistant *E. asburiae* isolate was diluted (1:10) in a prewarmed trypticase soy broth, allowed to culture in an antimicrobial-free medium for 2 h, and further cultured for 6 h with cefoxitin (2–50 mg/L) or imipenem (10–50 mg/L). β-Lactamase culture extracts were obtained after centrifugation and sonication, as detailed (*26*).

### Nucleic Acid Techniques and Conjugation

Genotype comparison of the imipenem-resistant *E. asburiae* strains was performed by using the random amplified polymorphism detection (RAPD) technique as described with primer 6MW (CCGACTCGAGNNNNNNATGTGG) and primers UBC 245 and UBC 282 (*26**,**28**,**29*). Transfer of the imipenem resistance marker from each imipenem-resistant *E. asburiae* isolate to *E. coli* DH10B was attempted by using the immobilization filter mating out technique, as described (*26*). Briefly, equal volume (0.1 mL) of overnight cultures of each *E. asburiae* isolate and *E. coli* DH10B were put onto a paper filter that was placed on an MH agar plate. Twenty-four hours later, the filter was removed, washed with water (0.2 mL), and the bacterial suspension was spread onto MH agar plates containing ampicillin (100 mg/L) and streptomycin (50 mg/L) for selecting transconjugants after 24 h (*26*).

Plasmid extraction was performed for each *E. asburiae* strain and their transconjugants and compared to reference plasmid sizes of *E. coli* NCTC 50192 by using the Kieser technique designed to extract large size plasmids (*30**,**31*). Whole-cell DNA of *Enterobacter* spp. reference strains and of an *E. asburiae* strain MS7 was extracted as described (*26*).

Southern hybridization of plasmid DNA (*26*) of the transconjugants was performed as described by the manufacturer with the ECL nonradioactive kit (Amersham, Les Ulis, France). An 818–bp internal probe for *bla*_IMI-1_ was obtained by using primers IMI-A (5´-ATAGCCATCCTTGTTTAGCTC-3´) and IMI-B (5´-TCTGCGATTACTTTATCCTC-3´) and standard polymerase chain reaction (PCR) amplification procedures (*5**,**26*).

Primers designed to hybridize to the ends of the *bla*_NmcA_, *bla*_IMI-1_, and *bla*_Sme-1/Sme-2_ genes were used for standard PCR amplification experiments (*5**,**7**,**8*) with plasmid DNA of each imipenem-resistant *E. asburiae* isolate and of their transconjugants as templates. Cloning experiments were then performed with *Bam*HI restricted whole-cell DNA of *E. asburiae* MS7 followed by ligation of DNA fragments into the *Bam*HI-site of cloning vector pGB2 (*32*). Recombinant plasmids were transformed by electroporation into *E. coli* DH10B electrocompetent cells (*26*). *E. coli* DH10B harboring recombinant plasmids was selected on MH agar plates containing ampicillin (100 mg/L) and streptomycin (100 mg/L).

DNA sequencing of both strands of PCR fragments amplified with the primers for *bla*_IMI-1_ and plasmid DNA of *E. asburiae* isolates as templates and of the cloned fragment of a recombinant plasmid was determined with an Applied Biosystems sequencer (ABI377). The nucleotide sequences and the deduced protein sequences were analyzed with software available on the Internet from the National Center for Biotechnology Information Web site (http://www.ncbi.nlm.nih.gov/BLAST).

## Results

### Bacterial Identification

Twenty-nine of the 30 imipenem-resistant isolates substantially hydrolyzed imipenem, i.e., 10.5 ± 1.6 U/mg of protein of culture extracts. These isolates were a single *Aeromonas hydrophila* isolate, 6 *Stenotrophomonas maltophilia* isolates known to naturally produce carbapenemases, and 22 *Enterobacter* spp. isolates identified as *E. asburiae* that were further analyzed.

As reported in Table 1, *E. asburiae* strains were isolated at different times from several rivers in the midwest. Other tested rivers had ampicillin-resistant isolates that were not imipenem-resistant (Figure). These rivers were Arkansas (Little Rock), Canadian (Oklahoma City), Hudson (New York), Chicago (Chicago), Colorado (Glenwood Springs), Missouri (Parkville), Cuyahoga (Cleveland), Mississippi (New Orleans, St. Louis), Ohio (Cincinnati, Louisville, Pittsburgh, Wheeling), Platte (Grand Island), Scioto (Columbus), Wabash (Terre Haute), and White (Indianapolis). RAPD analysis was then performed to compare all imipenem-resistant *E. asburiae* isolates. Using a series of different primers, this genotyping technique identified clonally indistinguishable *E. asburiae* isolates, although they were from various geographic origins (data not shown).

**Table 1:** Origin and date of isolation of imipenem-resistant *Enterobacter asburiae* environmental isolates

River (city)	Isolate	Date
Arkansas River (Wichita, KS)	*E. asburiae* AK1	September 1999
Kansas River (Topeka, KS)	*E. asburiae* K1–K5	September 2000
Des Moines River (Des Moines, IA)	*E. asburiae* DM1–DM8	August 2001
Mississippi River (Minneapolis, MN)	*E. asburiae* MS1–MS8	August 2001

**Figure:**
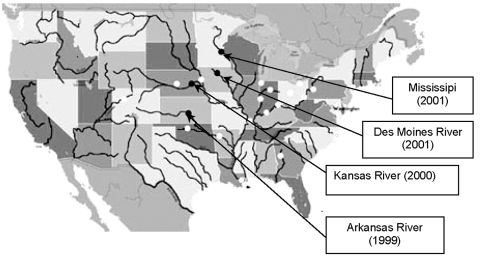
Sites of isolation of IMI-2–producing *Enterobacter asburiae* isolates (black circles) and ampicillin-resistant, gram-negative rods (white circles).

### β-Lactam Resistance Marker

The imipenem resistance marker was transferred from each imipenem-resistant *E. asburiae* isolate to *E. coli* DH10B by conjugation. Plasmid analysis identified a 66-kb plasmid (pNat) from cultures of each imipenem-resistant *E. asburiae* isolate, whereas this plasmid was not isolated from *E. cloacae* and *E. asburiae* reference strains (data not shown). PCR experiments with primers for the *bla*_IMI-1_ gene were positive with plasmid DNA of each *E. asburiae* isolate and transconjugants as templates, whereas primers designed to amplify *bla*_NmcA_ and *bla*_Sme-1/Sme-2_ failed to give PCR product. The Southern blot analysis confirmed that the *bla*_IMI_-like gene was located on the natural plasmid pNat (data not shown).

Sequencing PCR products with primers hybridizing at the ends of the *bla*_IMI-1_ gene and plasmid DNA of each imipenem-resistant *E. asburiae* isolate identified the same β-lactamase IMI-2 in all cases. This novel enzyme had 2 amino acid substitutions (tyrosine to histidine at position Ambler 105 and asparagine to aspartic acid at position Ambler 35) compared to the chromosomally encoded carbapenemase IMI-1 (*5*). β-Lactamase IMI-1 had been isolated from an *E. cloacae* isolate from Minnesota close to locations where IMI-2–producing isolates have been found (*5*). However, the *bla*_IMI-2_ gene was not just a point-mutant derivative of the *bla*_IMI-1_ gene, since these genes differ by 11 nucleotide substitutions. β-Lactamase IMI-2 was also related to NmcA (97% amino acid identity) (*8*).

Cloning *Bam*HI-restricted DNA of whole-cell DNA of *E. asburiae* MS7 gave recombinant plasmid pIMI-2 that had a 5.5-kb insert that allowed identification of the surrounding sequence of the *bla*_IMI-2_ gene. A gene encoding a LysR-type regulator named IMIR-2 was found just upstream of *bla*_IMI-2_. It shared 95% amino acid identity with IMIR-1, which is located upstream of the *bla*_IMI-1_ gene (*5*). The surrounding sequences of *bla*_IMI-2_ shared significant nucleotide identity with transposable elements. Part of an open reading frame that shared 97% nucleotide identity with that of the transposase gene *tnpA* of the transposon Tn*2501* (Tn*3* family) was identified downstream of *bla*_IMI-2_ (*33*). Upstream of *imiR-2*, a 142-bp sequence shared 76% nucleotide identity with part of the insertion sequence IS*2*.

### Susceptibility Testing and Expression of Resistance

MICs of several β-lactams, including carbapenems for the IMI-2–positive *E. asburiae* MS7 and for *E. coli* DH10B expressing the *bla*_IMI-2_ gene were high (Table 2). The MICs of β-lactams for all imipenem-resistant clinical isolates were identical (data not shown). Much higher level of resistance to aztreonam than to expanded-spectrum cephalosporins was found for the IMI-2–positive strains, as reported for the other producers of class A carbapenemases (*2*). The activity of β-lactamase IMI-2 was partially inhibited by clavulanate and tazobactam. Induction studies showed increase of β-lactamase expression from 17- to 30-fold (170 to 300 U/mg of protein) (for each *E. asburiae* isolate when imipenem (50 mg/L) and cefoxitin (50 mg/L) were used as inducers, respectively. These induction results were consistent with location and functionality of a LysR-type regulator gene upstream of the *bla*_IMI-2_ gene in the imipenem-resistant *E. asburiae* isolates. No other antimicrobial resistance marker was carried by natural plasmid pNat.

**Table 2:** MICs (mg/L) of β-lactams for several carbapenemase producers and reference strain *Escherichia coli* DH10B

β-Lactam(s)*	*Enterobacter asburiae* MS7†	*E. cloacae* 1413B†	*Escherichia coli* DH10B (pNat)‡	*E. coli* DH10B (pIMI-2)‡	*E. coli* DH10B
Amoxicillin	>512	>512	>512	>512	4
Amoxicillin + CLA	>512	>512	>512	>512	4
Ticarcillin	128	>256	128	256	4
Ticarcillin + CLA	16	>256	16	32	4
Piperacillin	16	>256	8	128	2
Piperacillin + TZB	4	>256	2	16	2
Cephalothin	512	>256	64	512	4
Cefotaxime	0.06	1	0.06	1	0.06
Ceftazidime	0.12	2	0.06	0.5	0.25
Aztreonam	4	8	4	64	0.12
Imipenem	>64	>64	16	>64	0.06
Meropenem	32	4	2	32	0.06

*CLA, clavulanic acid at a fixed concentration of 2 mg/L; TZB, tazobactam at a fixed concentration of 4 mg/L. †*Enterobacter asburiae* MS7 produces acquired β-lactamase IMI-2, whereas *E. cloacae* 1413B produces acquired β-lactamases TEM-1 and IMI-1 (*5*). ‡Natural plasmid pNat harbors the *bla*_IMI-2_ gene, whereas pIMI-2 is a recombinant plasmid that has the same β-lactamase gene.

## Discussion

This report indicates that several U.S. rivers may be a reservoir for broad-spectrum carbapenemases. Here, we report a novel clavulanic-acid inhibited Ambler class A β-lactamase IMI-2 that has an usual spectrum of hydrolysis for this type of β-lactamase, including penicillins, carbapenems, and aztreonam (*2*). β-Lactamase IMI-2 is closely related to several Ambler class A carbapenemases whose genes are chromosomally located, including *bla*_IMI-1_ and *bla*_NmcA_, and found in several clinical isolates (*5**,**8*). While this work was in progress, a clinical case of an NmcA-producing *E. cloacae* isolate was reported from Seattle (*34*). An extended epidemiologic survey identified Sme-1 type–producing *Serratia marcescens* isolates from the West Coast to the East Coast, which indicates that these isolates may also represent a reservoir for carbapenemases in *Enterobacteriaceae* (*9*). Thus, identification of carbapenemase genes in enterobacterial strains from rivers may have clinical importance.

In the present study, the β-lactamase gene was plasmid-encoded and was adjacent to mobile sequences that may play an additional role in gene transfer. The *E. asburiae* isolates were clonally related and may correspond to a single clone, although they were obtained from distantly related midwestern rivers. The reason for the presence of these antimicrobial-resistant strains in this region is unknown. Taking into account the small number of specimens withdrawn from the rivers and the selection technique for imipenem-resistant isolates (ampicillin- and not imipenem-containing plates), the prevalence of carbapenemase-producing enterobacterial strains may be high in the environment, at least in the Midwest.

Cloning experiments led to identification of a regulatory gene from an *E. asburiae* strain (found in the other *E. asburiae* strains as well [data not shown]) that explained inducibility of carbapenemase expression. Whatever the level of imipenem resistance is, failure of an imipenem-containing regimen may occur when treating infections caused by similar carbapenemase-producing strains, as deduced from results obtained with an animal model of pneumonia (*35*). Finally, this study raises the question of the importance of this reservoir in *Enterobacteriaceae* as well as the origin of this plasmid-located carbapenemase gene that may be transferred among other enterobacterial pathogens.
